# Eight new *Arthrinium* species from China

**DOI:** 10.3897/mycokeys.34.24221

**Published:** 2018-05-03

**Authors:** Mei Wang, Xiao-Ming Tan, Fang Liu, Lei Cai

**Affiliations:** 1 State Key Laboratory of Mycology, Institute of Microbiology, Chinese Academy of Sciences, Beijing 100101, PR China; 2 University of Chinese Academy of Sciences, Beijing 100049, PR China; 3 College of Navel Orange, Gannan Normal University, Ganzhou, Jiangxi, 341000, PR China

**Keywords:** Ascomycota, Morphology, Phylogeny, Systematics, Taxonomy

## Abstract

The genus *Arthrinium* includes important plant pathogens, endophytes and saprobes with a wide host range and geographic distribution. In this paper, 74 *Arthrinium* strains isolated from various substrates such as bamboo leaves, tea plants, soil and air from karst caves in China were examined using a multi-locus phylogeny based on a combined dataset of ITS rDNA, TEF1 and TUB2, in conjunction with morphological characters, host association and ecological distribution. Eight new species were described based on their distinct phylogenetic relationships and morphological characters. Our results indicated a high species diversity of *Arthrinium* with wide host ranges, amongst which, Poaceae and Cyperaceae were the major host plant families of *Arthrinium* species.

## Introduction

*Arthrinium* Kunze is an anamorph-typified genus, which has been traditionally linked to the teleomorph-typified genus *Apiospora* Sacc. (Ellis 1971, Seifert et al. 2011). It is strikingly different from other anamorphic genera for the presence of basauxic conidiophores (Hughes 1953, Minter 1985). The traditional generic circumscription of *Arthrinium* was primarily based on morphological characters (e.g. conidial shape, conidiophores, sterile cells and the presence of setae) but has been regarded as too narrow (Ellis 1971, Minter 1985, Crous et al. 2013). It is now recognised that, at the generic level, conidial shape and the presence of setae are not reliable characters to infer phylogenetic relationships (Crous et al. 2013). For example, *Arthrinium* was regarded as being different from *Cordella* Speg. (1886) by the absence of setae amongst the clusters of specialised hyphae and different from *Pteroconium* Sacc. (1892) by the absence of sporodochia and pseudoparenchyma (Minter 1985). However, both genera have been reduced to the generic synonyms of *Arthrinium*, based on molecular phylogenetic data (Crous et al. 2013).

*Arthrinium* species are geographically widely distributed in various hosts. Many species of *Arthrinium* are associated with plants as endophytes or saprobes, as well as plant pathogens on some important ornamentals, e.g. *A. phaeospermum* causing culm rot on *Phyllostachys viridis* (Li et al. 2016); *A. arundinis* causing brown culm streak of *Phyllostachys praecox* (Chen et al. 2014). Moreover, *A. phaeospermum* has been reported for causing cutaneous infections of humans (Rai 1989, Zhao et al. 1990, de Hoog et al. 2000, Crous et al. 2013). Many *Arthrinium* species are also known to produce bioactive compounds with pharmacological and medicinal applications, such as *A. arundinis* and *A. saccharicola* isolated from a brown alga *Sargassum* sp., with good antifungal activities against some plant pathogenic fungi (Hong et al. 2015). *Arthrinium saccharicola*, *A. sacchari* and *A. phaeospermum* isolated from *Miscanthus* sp. are known to produce industrially important enzymes (Shrestha et al. 2015).

In this paper, eight new *Arthrinium* species are described and characterised based on morphological characters and phylogeny inferred from the combined ITS rDNA, TEF1 and TUB2 sequences dataset. Comparisons were made with morphologically similar and phylogenetically related species. Fungus-host distribution of *Arthrinium* species are summarised based on data from literature and this study.

## Materials and method

### Isolates

Diseased and healthy tissues of bamboo leaves and other plant hosts were collected from six provinces or municipalities in China (Chongqing, Guangxi, Guangdong, Guizhou, Jiangxi, Hunan). Tissue pieces (5 mm × 5 mm) were taken from the margin of leaf lesions and the surface sterilised with 75% ethanol for 1 min, 5% NaClO for 30 s, followed by rinsing in sterile distilled water for 1 min. The pieces were dried with sterilised paper towels and then placed on 1/4 PDA (potato dextrose agar) (Cai et al. 2009).

All cultures were preserved in the LC culture collection (personal culture collection of Lei Cai housed in the Institute of Microbiology, Chinese Academy of Sciences). Type specimens were deposited in Mycological Herbarium of the Institute of Microbiology, Chinese Academy of Sciences, Beijing, China (HMAS), with ex-type living cultures deposited in China General Microbiological Culture Collection Center (CGMCC). Taxonomic information of the new taxa was deposited in MycoBank (www.MycoBank.org; Crous et al. 2004).

### Morphology

Cultures were incubated on PDA for 7 d at 25 °C to measure the growth rates and on 2% malt agar with bamboo leaves to enhance sporulation. Morphological descriptions were based on cultures sporulating on MEA (malt extract agar) medium at room temperature (ca. 25 °C). Shape and size of microscopic structures were observed using a light microscope and colonies were assessed according to the colour charts of Rayner (1970). At least 50 conidiogenous cells and conidia were measured to calculate the mean size.

### DNA extraction, PCR amplification and sequencing

Fresh fungal mycelia were taken from 7-d-old cultures growing on PDA and ground with the organisation disruptor FastPrep-48. Genomic DNA was extracted following the modified CTAB protocol as described in Guo et al. (2000).

Phylogenetic analyses were conducted using partial sequences of three loci, 5.8S nuclear ribosomal gene with the two flanking transcribed spacers (ITS), part of the translation elongation factor 1-alpha (TEF1) and beta-tubulin (TUB2). The ITS locus was amplified using the primer pair ITS1/ITS4 (Vilgalys and Hester 1990, White et al. 1990); TEF1 using EF1-728F/ EF-2 (O’Donnell et al. 1998, Carbone and Kohn 1999); and TUB2 using T1 (O’Donnell and Cigelnik 1997) and Bt-2b (Glass and Donaldson 1995).

PCR was performed in a 25 ml reaction containing 18.95 µl double distilled water, 2.5 µl 10 × PCR buffer, 0.3 µl dNTP mix (2.5 mM), 1 µl of each primer (10 mM), 1 µl DNA template and 0.25 µl Taq DNA polymerase (Genstar). The annealing temperatures were adjusted to 52 °C for ITS and TUB2, and 56 °C for TEF1. Purification and sequencing of the PCR amplicons were done by SinoGenoMax, Beijing.

### Phylogenetic analysis

**Figure 1.**
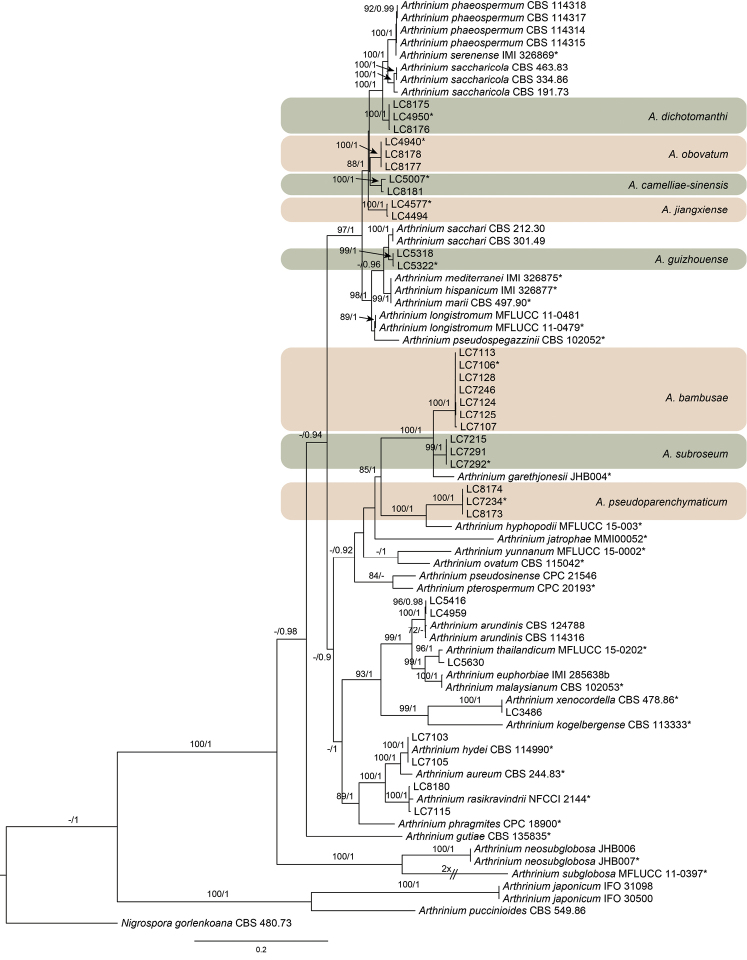
Phylogenetic tree based on the combined ITS, TEF1 and TUB2 sequences alignment generated from a Maximum likelihood phylogenetic analysis. Bootstrap support values (>70%) and posterior probabilities (>0.9) are given at the nodes (ML/PP). The tree is rooted with *Nigrospora gorlenkoana* CBS 480.73. The novel species were highlighted (* indicates the ex-type cultures).

Sequences generated from the forward and reverse primers were used to obtain consensus sequences using MEGA v. 6.0 (Tamura et al. 2013). The concatenated tree was inferred based ITS, TUB2 and TEF1 sequences (Figure 1) using Bayesian and Maximum-likelihood analyses. Sequences were aligned using an online version of MAFFT v. 7 (available at http://mafft.cbrc.jp/alignment/server/). Ambiguous regions were excluded from the analyses and gaps were treated as missing data. Maximum-likelihood (ML) analysis was performed in RAxML v. 7.2.6 (Stamatakis and Alachiotis 2010), employing GTR models of evolution settings of the programme and bootstrap support obtained by running 1000 pseudo replicates. Maximum Likelihood bootstrap values (ML) equal to or greater than 70% are given above each node.

Bayesian analysis was conducted using MrBayes v. 3.2.1 (Ronquist et al. 2012) and the best nucleotide substitution model for each locus was calculated with jModelTest v. 2.1.4 (Posada 2008). Posterior probabilities (PP) (Zhaxybayeva and Gogarten 2002) were determined by Markov Chain Monte Carlo sampling (MCMC) under the estimated model of evolution. Four simultaneous Markov chains were run for 10 million generations and trees were sampled every 1000 generations. The run was stopped automatically when the average standard deviation of split frequencies fell below 0.01. The first 25% trees, which represented the burn-in phase of the analyses, were discarded and the remaining trees were used for calculating PP in the majority rule consensus tree. Sequences generated in this study were deposited in GenBank (Table 1) and the final matrices used for the phylogenetic analyses in TreeBASE (www.treebase.org; accession number: 21341).

**Table 1.** Strains included in the phylogenetic analyses.

Speices	Strain numbers^1^	Hosts	Countries	GenBank accessions		
				ITS	TUB	TEF
*Arthirinium arundinis*	CBS 114316	Leaf of *Hordeum vulgare*	Iran	KF144884	KF144974	KF145016
	CBS 124788	Living leaves of *Fagus sylvatica*	Switzerland	KF144885	KF144975	KF145017
	**LC4477**	**Unknow host**	**China**	**KY494688**	**KY705159**	**KY705087**
	**LC4493**	***Phyllostachys* sp.**	**China**	**KY494689**	**KY806202**	**KY705088**
	**LC4650**	***Osmanthus* sp.**	**China**	**KY494695**	**KY705165**	**KY705094**
	**LC4951**	***Dichotomanthus tristaniaecarpa***	**China**	**KY494698**	**KY705168**	**KY705097**
	**LC4959**	***Bothrocaryum controversum***	**China**	**KY494699**	**KY705169**	**KY705098**
	**LC5311**	**Air in karst ca**ve	**China**	**KY494706**	**KY705175**	**KY705105**
	**LC5312**	**Air in karst cave**	**China**	**KY494707**	**KY705176**	**KY705106**
	**LC5332**	**Air in karst cave**	**China**	**KY494710**	**KY705179**	**KY705109**
	**LC5394**	**Soil in karst cave**	**China**	**KY494711**	**KY705180**	**KY705110**
	**LC5416**	**Water in karst cave**	**China**	**KY494712**	**KY705181**	**KY705111**
	**LC7118**	**Leaf of bamboo**	**China**	**KY494723**	**KY705191**	**KY705120**
	**LC7122**	**Leaf of bamboo**	**China**	**KY494726**	**KY705194**	**KY705123**
	**LC7160**	**Leaf of bamboo**	**China**	**KY494738**	**KY705206**	**KY705134**
	**LC7211**	**Leaf of bamboo**	**China**	**KY494739**	**KY705207**	**KY705135**
	**LC7216**	**Leaf of bamboo**	**China**	**KY494741**	**KY705209**	**KY705137**
	**LC7218**	**Leaf of bamboo**	**China**	**KY494742**	**KY705210**	**KY705138**
	**LC7243**	**Leaf of bamboo**	**China**	**KY494744**	**KY705212**	**KY705140**
	**LC7252**	**Leaf of bamboo**	**China**	**KY494747**	**KY705215**	**KY705143**
	**LC7277**	**Leaf of bamboo**	**China**	**KY494750**	**KY705218**	**KY705146**
*A. aureum*	CBS 244.83*	Air	Spain	AB220251	KF144981	KF145023
***A. bambusae***	LC7106* = CGMCC 3.18335	**Leaf of bamboo**	**China**	**KY494718**	**KY705186**	**KY806204**
	**LC7107**	**Leaf of bamboo**	**China**	**KY494719**	**KY705187**	**KY705117**
	**LC7113**	**Leaf of bamboo**	**China**	**KY494720**	**KY705188**	**KY806205**
	**LC7124**	**Leaf of bamboo**	**China**	**KY494727**	**KY705195**	**KY806206**
	**LC7125**	**Leaf of bamboo**	**China**	**KY494728**	**KY705196**	**KY705124**
	**LC7128**	**Leaf of bamboo**	**China**	**KY494730**	**KY705198**	**KY705126**
	**LC7246**	**Leaf of bamboo**	**China**	**KY494745**	**KY705213**	**KY705141**
***A. camelliae-sinensis***	LC5007* = CGMCC 3.18333	***Camellia sinensis***	**China**	**KY494704**	**KY705173**	**KY705103**
	**LC8181**	***Brassica capestris***	**China**	**KY494761**	**KY705229**	**KY705157**
***A. dichotomanthi***	LC4950* = CGMCC 3.18332	***Dichotomanthus tristaniaecarpa***	**China**	**KY494697**	**KY705167**	**KY705096**
	**LC8175**	***Dichotomanthus tristaniaecarpa***	**China**	**KY494755**	**KY705223**	**KY705151**
	**LC8176**	***Dichotomanthus tristaniaecarpa***	**China**	**KY494756**	**KY705224**	**KY705152**
*A. euphorbiae*	IMI 285638b	*Bambusa* sp.	Bangladesh	AB220241	AB220288	–
***A. guizhouense***	**LC5318**	**Air in karst cave**	**China**	**KY494708**	**KY705177**	**KY705107**
	LC5322* =CGMCC3.18334	**Air in karst cave**	**China**	**KY494709**	**KY705178**	**KY705108**
*A. gutiae*	CBS 135835	Gut of a grasshopper	India	KR011352	KR011350	KR011351
*A. hispanicum*	IMI 326877*	Maritime sand	Spain	AB220242	AB220289	–
*A. hydei*	CBS 114990*	Culms of *Bambusa tuldoides*	Hong Kong	KF144890	KF144982	KF145024
	**LC7103**	**Leaf of bamboo**	**China**	**KY494715**	**KY705183**	**KY705114**
	**LC7105**	**Leaf of bamboo**	**China**	**KY494717**	**KY705185**	**KY705116**
*A. hyphopodii*	MFLUCC 15-0003*	Culms of *Bambusa tuldoides*	Thailand	KR069110	–	–
*A. japonicum*	IFO 30500	*Carex despalata* (dead leaf)	Japan	AB220262	AB220309	–
	IFO 31098	*Carex despalata* (leaf)	Japan	AB220264	AB220311	–
*A. garethjonesii*	KUMCC 16-0202	Dead culms of bamboo	China	KY356086	–	–
*A. jatrophae*	MMI 00052* = MCC 1014	Healthy petiole of *Jatropha podagrica*	India	JQ246355	–	–
***A. jiangxiense***	**LC2831**	**Leaf of bamboo**	**China**	**KY494686**	**KY80620106201**	**KY705085**
	**LC4494**	***Phyllostachys* sp.**	**China**	**KY494690**	**KY705160**	**KY705089**
	**LC4541**	***Maesa* sp.**	**China**	**KY494691**	**KY705161**	**KY705090**
	**LC4547**	***Machilus* sp.**	**China**	**KY494692**	**KY705162**	**KY705091**
	LC4577* = CGMCC 3.18381	***Maesa* sp.**	**China**	**KY494693**	**KY705163**	**KY705092**
	**LC4578**	***Camellia sinensis***	**China**	**KY494694**	**KY705164**	**KY705093**
	**LC4993**	***Phyllostachys* sp.**	**China**	**KY494700**	**KY806203**	**KY705099**
	**LC4997**	***Phyllostachys* sp.**	**China**	**KY494701**	**KY705170**	**KY705100**
	**LC5001**	***Phyllostachys* sp.**	**China**	**KY494702**	**KY705171**	**KY705101**
	**LC5004**	***Phyllostachys* sp.**	**China**	**KY494703**	**KY705172**	**KY705102**
	**LC5015**	***Imperata cylindrica***	**China**	**KY494705**	**KY705174**	**KY705104**
***A. jiangxiense***	**LC7104**	**Leaf of bamboo**	**China**	**KY494716**	**KY705184**	**KY705115**
	**LC7154**	**Leaf of bamboo**	**China**	**KY494736**	**KY705204**	**KY705132**
	**LC7156**	**Leaf of bamboo**	**China**	**KY494737**	**KY705205**	**KY705133**
	**LC7275**	**Leaf of bamboo**	**China**	**KY494749**	**KY705217**	**KY705145**
*A. kogelbergense*	CBS 113333*	Dead culms of Restionaceae	South Africa	KF144892	KF144984	KF145026
*A. longistromum*	MFLUCC 11-0481*	Decaying bamboo culms	Thailand	KU940141	–	–
	MFLUCC 11-0479	Decaying bamboo culms	Thailand	KU940142	–	–
*A. malaysianum*	CBS 102053*	*Macaranga hullettii* stem colonised by ants	Malaysia	KF144896	KF144988	KF145030
*A. marii*	CBS 497.90*	Air	Spain	AB220252	KF144993	KF145035
*A. mediterranei*	IMI 326875*	Air	Spain	AB220243	AB220290	–
***A. mytilomorphum***	DAOM 214595*	**Dead blades of *Andropogon* sp.**	**India**	**KY494685**	–	–
*A. neosubglobosa*	JHB006	Dead culms of bamboo	China	KY356089	–	–
	KUMCC 16-0203	Dead culms of bamboo	China	KY356090	–	–
***A. obovatum***	LC4940* = CGMCC 3.18331	***Lithocarpus* sp.**	**China**	**KY494696**	**KY705166**	**KY705095**
	**LC8177**	***Lithocarpus* sp.**	**China**	**KY494757**	**KY705225**	**KY705153**
	**LC8178**	***Lithocarpus* sp.**	**China**	**KY494758**	**KY705226**	**KY705154**
*A. ovatum*	CBS 115042*	*Arundinaria hindsii*	Hong Kong	KF144903	KF144995	KF145037
*A. paraphaeospermum*	MFLU 16-1974	Dead clumps of *Bambusa* sp.	Thailand	KX822128	–	–
*A. phaeospermum*	CBS 114314	Leaf of *Hordeum vulgare*	Iran	KF144904	KF144996	KF145038
	CBS 114315	Leaf of *Hordeum vulgare*	Iran	KF144905	KF144997	KF145039
	CBS 114317	Leaf of *Hordeum vulgare*	Iran	KF144906	KF144998	KF145040
	CBS 114318	Leaf of *Hordeum vulgare*	Iran	KF144907	KF144999	KF145041
*A. phragmites*	CPC18900*	Culms of *Phragmites australis*	Italy	KF144909	KF145001	KF145043
***A. pseudoparenchymaticum***	LC7234* = CGMCC 3.18336	**Leaf of bamboo**	**China**	**KY494743**	**KY705211**	**KY705139**
	**LC8173**	**Leaf of bamboo**	**China**	**KY494753**	**KY705221**	**KY705149**
	**LC8174**	**Leaf of bamboo**	**China**	**KY494754**	**KY705222**	**KY705150**
*A. pseudosinense*	CPC 21546*	Leaf of bamboo	The Netherlands	KF144910	–	KF145044
*A. pseudospegazzinii*	CBS 102052*	*Macaranga hullettii* stem colonised by ants	Malaysia	KF144911	KF145002	KF145045
*A. pterospermum*	CPC 20193*	Leaf lesion of *Machaerina sinclairii*	Australia	KF144913	KF145004	KF145046
*A. puccinioides*	CBS 549.86	Leaf of *Lepidosperma gladiatum*	Germany	AB220253	AB220300	–
*A. rasikravindrii*	CBS 337.61	*Cissus* sp.	The Netherlands	KF144914	–	–
	CPC 21602	Rice	Thailand	KF144915	–	–
	MFLUCC 15-0203	Dead bamboo culms	Thailand	KU940143	–	–
	MFLUCC 11-0616	Dead bamboo culms	Thailand	KU940144	–	–
	NFCCI 2144*	Soil	Svalbard	JF326454	–	–
	**LC5449**	**Soil in karst cave**	**China**	**KY494713**	**KY705182**	**KY705112**
	**LC7115**	**Leaf of bamboo**	**China**	**KY494721**	**KY705189**	**KY705118**
	**LC7117**	**Leaf of bamboo**	**China**	**KY494722**	**KY705190**	**KY705119**
	**LC7119**	**Leaf of bamboo**	**China**	**KY494724**	**KY705192**	**KY705121**
	**LC7120**	**Leaf of bamboo**	**China**	**KY494725**	**KY705193**	**KY705122**
	**LC7126**	**Leaf of bamboo**	**China**	**KY494729**	**KY705197**	**KY705125**
	**LC7129**	**Leaf of bamboo**	**China**	**KY494731**	**KY705199**	**KY705127**
	**LC7135**	**Leaf of bamboo**	**China**	**KY494732**	**KY705200**	**KY705128**
	**LC7139**	**Leaf of bamboo**	**China**	**KY494733**	**KY705201**	**KY705129**
	**LC7141**	**Leaf of bamboo**	**China**	**KY494734**	**KY705202**	**KY705130**
	**LC7142**	**Leaf of bamboo**	**China**	**KY494735**	**KY705203**	**KY705131**
	**LC7251**	**Leaf of bamboo**	**China**	**KY494746**	**KY705214**	**KY705142**
	**LC7254**	**Leaf of bamboo**	**China**	**KY494748**	**KY705216**	**KY705144**
	**LC8179**	***Brassica capestris***	**China**	**KY494759**	**KY705227**	**KY705155**
	**LC8180**	***Brassica capestris***	**China**	**KY494760**	**KY705228**	**KY705156**
*A. sacchari*	CBS 212.30	*Phragmites australis*	United Kingdom	KF144916	KF145005	KF145047
	CBS 301.49	Bamboo	Indonesia	KF144917	KF145006	KF145048
*A. saccharicola*	CBS 191.73	Air	The Netherlands	KF144920	KF145009	KF145051
	CBS 334.86	Dead culms of *Phragmites australis*	France	AB220257	KF145010	KF145052
	CBS 463.83	Dead culms of *Phragmites australis*	The Netherlands	KF144921	KF145011	KF145053
*A. serenense*	IMI 326869*	Food, pharmaceutical excipients, atmosphere and home dust	Spain	AB220250	AB220297	–
*A. subglobosum*	MFLUCC 11-0397*	Dead bamboo culms	Thailand	KR069112	–	–
***A. subroseum***	**LC7215**	**Leaf of bamboo**	**China**	**KY494740**	**KY705208**	**KY705136**
	**LC7291**	**Leaf of bamboo**	**China**	**KY494751**	**KY705219**	**KY705147**
	LC7292* =CGMCC3.18337	**Leaf of bamboo**	**China**	**KY494752**	**KY705220**	**KY705148**
*A. thailandicum*	MFLUCC 15-0202*	Dead bamboo culms	Thailand	KU940145	–	–
	**LC5630**	**Rotten wood**	**China**	**KY494714**	**KY806200**	**KY705113**
*A. xenocordella*	CBS 478.86*	Soil from roadway	Zimbabwe	KF144925	KF145013	KF145055
	**LC3486**	***Camellia sinensis***	**China**	**KY494687**	**KY705158**	**KY705086**
*A. yunnanum*	MFLUCC 15-0002*	Decaying bamboo culms	China	KU940147	–	–
*N. gorlenkoana*	CBS 480.73	*Vitis vinifera*	Kazakhstan	KX986048	KY019456	KY019420

^*^= type strains, strains and sequences generated in this study are shown in **bold.**

^1^ CBS: Westerdijk Fungal Biodiversity Institute, Utrecht, The Netherlands; CGMCC: China General Microbiological Culture Collection; CPC: Culture collection of Pedro Crous, housed at the Westerdijk Fungal Biodiversity Institute; DAOM: Canadian Collection of Fungal Cultures, Ottawa, Canada; DSM: Deutsche Sammlung von Mikroorganismen und Zellkulturen GmbH, Braunschweig, Germany; IMI: Culture collection of CABI Europe UK Centre, Egham, UK; IFO: Institute for Fermentation, Osaka; LC: Working collection of Lei Cai, housed at CAS, China; MFLUCC: Mae Fah Luang University Culture Collection, Chiang Rai, Thailand; MCC: Microbial Culture Collection of India; NFCCI: National Fungal Culture Collection of India.

### Fungus-host distribution of *Arthrinium* species

To determine the distribution of *Arthrinium* species on host/substrate, the number of species occurred on each host (based on family level) was counted based on data from this study, relevant literature and the USDA fungal database (https://nt.ars-grin.gov/fungaldatabases/). The proportion account for the known 66 species in *Arthrinium* (Index Fungorum) was illustrated in a histogram. Four species with an unknown host range were not included in this analysis.

## Results

### Phylogeny

The combined ITS, TUB2 and TEF1 dataset contained 75 strains, with *Nigrospora gorlenkoana* CBS 480.73 as the out group. For the Bayesian analyses, the best-fit models TrN+I+G, GTR+I+G, HKY+I+G were selected for ITS, TUB2 and TEF1 loci, respectively. The ML analysis showed the same tree topology as that obtained in the Bayesian analysis. All the *Arthrinium* strains in this study separated into 13 clades, representing five known (*A. arundinis*, *A. hydei*, *A. rasikravindrii*, *A. thailandicum*, *A. xenocordella*) and eight new species (Figure 1). The eight new species clustered in distinct clades with high bootstrap supports (Figure 1). Phylogenetic analyses based on an individual locus were also conducted (not shown) and the generated trees are similar to the one generated from the combined multi-locus dataset (Figure 1).

### Host associated with *Arthrinium* species

**Figure 2.**
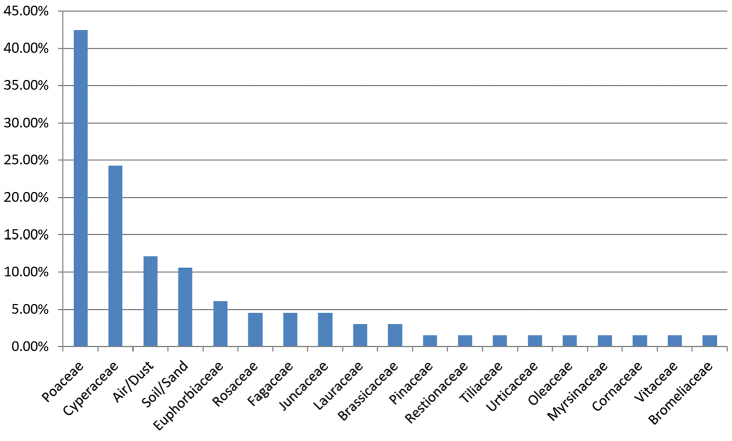
A Histogram to show fungus-host distribution of *Arthrinium* species.

The histogram in Figure 2 shows that *Arthrinium* species were widely distributed amongst 17 plant families, including Brassicaceae, Bromeliaceae, Cornaceae, Cyperaceae, Euphorbiaceae, Fagaceae, Juncaceae, Lauraceae, Myrsinaceae, Oleaceae, Pinaceae, Poaceae, Restionaceae, Rosaceae, Tiliaceae, Urticaceae and Vitaceae. *Arthrinium* species were also isolated from air, dust, soil and sand. The proportion of species occurring on each host family was assessed (Figure 2). Poaceae and Cyperaceae were the major host families for *Arthrinium*, which accounted for 42.42% and 24.24% of species in *Arthrinium* respectively.

## Taxonomy

### Arthrinium bambusae

Taxon classificationFungiXylarialesApiosporaceae

M. Wang & L. Cai sp. nov.

824906

Figure 3

#### Type.

CHINA, Guangdong Province, on bamboo leaves, 10 Jul. 2016, D.W. Xiao, (holotype: HMAS 247187; culture ex-type: CGMCC 3.18335 = LC7106).

#### Etymology.

Named after the host of the holotype.

#### Description.

Hyphae hyaline, branched, septate, 1.5–5.0 μm diam. Conidiophores reduced to conidiogenous cells. Conidiogenous cells erect, aggregated in clusters on hyphae, hyaline to pale brown, smooth, doliiform to ampulliform, or lageniform, 4.0–12.0 × 3.0–7.0 μm (*x̄* = 6.6 ± 1.8 × 4.8 ± 0.9, n = 30). Conidia olivaceous to brown, smooth to finely roughened, subglobose to ellipsoid, 11.5–15.5 × 7.0–14.0 μm (*x̄* = 13.2 ± 0.8 × 11.4 ± 1.2, n = 50).

#### Culture characteristics.

On PDA, colonies flat, spreading, margin circular, with abundant aerial mycelia, surface and reverse white to grey. On MEA, colonies flat, spreading, surface and reverse brown to black.

#### Additional specimens examined.

CHINA, Jiangxi Province, on bamboo leaves, 10 Jul. 2016, Q. Xiong, living culture LC7246; Guangdong Province, on bamboo leaves, 10 Jul. 2016, D.W. Xiao, living culture LC7107; ibid. living culture LC7113; ibid. living culture LC7124; ibid. living culture LC7125; ibid. living culture LC7128.

#### Notes.

Seven strains representing *A. bambusae* clustered in a well-supported clade closely related to *A. subroseum* (98% sequence similarity in ITS; 92% in TUB2; 96% in TEF1). *Arthrinium bambusae* differs from *A. subroseum* in the morphology of conidiophore (reduced to conidiogenous cells in *A. bambusae* vs. erect or ascending, clustered in groups in *A. subroseum*). Moreover, *A. bambusae* does not produce pigment on the PDA.

**Figure 3.**
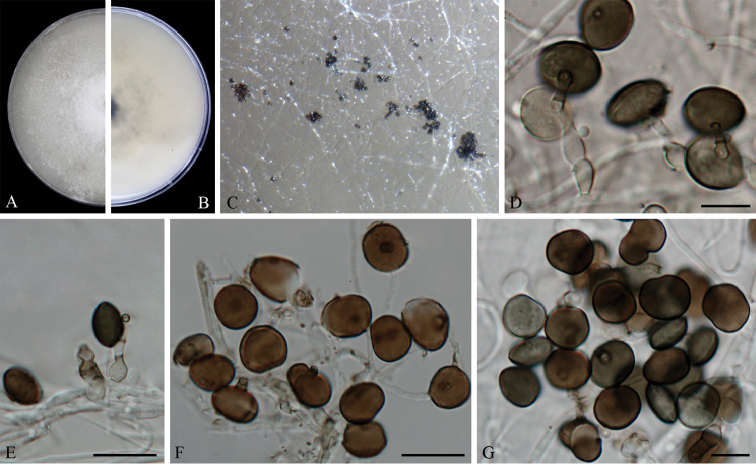
*Arthrinium bambusae* (from ex-holotype strain CGMCC 3.18335) **A–B** 7 d old cultures on PDA **C** Colony on MEA producing conidia masses **D–F** Conidiogenous cells giving rise to conidia **G** Conidia. Scale bars = 10 μm.

### Arthrinium camelliae-sinensis

Taxon classificationFungiXylarialesApiosporaceae

M. Wang, F. Liu & L. Cai sp. nov.

824907

Figure 4

#### Type.

CHINA, Jiangxi Province, on *Camellia sinensis*, 22 Apr. 2013, Q. Chen, (holotype: HMAS 247186; culture ex-type: CGMCC 3.18333 = LC5007).

#### Etymology.

Named with the host plant of the type.

#### Description.

Hyphae hyaline, branched, septate, 2.0–4.5 μm diam. Conidiophores reduced to conidiogenous cells. Conidiogenous cells erect, aggregated in clusters, hyaline to pale brown, smooth, doliiform to ampulliform, 4.0–9.5 × 3.0–6.0 μm (*x̄* = 6.1 ± 1.4 × 4.4 ± 0.9, n = 30). Conidia brown to dark brown, smooth, globose to subglobose, 9.0–13.5 × 7.0–12.0 μm (*x̄* = 11.1 ± 0.9 × 10.1 ± 1.0, n = 50).

#### Culture characteristics.

On PDA, colonies flat, margin circular, initially white, becoming greyish on surface, reaching 9 cm in 7 days at 25 °C. On MEA, with sparse aerial mycelia, surface dirty white, reverse pale luteous.

#### Other specimens.

CHINA, Hubei Province, on *Brassica campestris*, 31 Mar. 2016, Y.Z. Zhao, living culture LC8181 = LF1498.

#### Notes.

Two strains representing *A. camelliae-sinensis* clustered in a well-supported clade and appeared closely related to *A. jiangxiense* (97% sequence similarity in ITS; 94% in TUB2; 94% in TEF1) and *A. obovatum* (98% sequence similarity in ITS; 95% in TUB2; 93% in TEF1). While *A. camelliae-sinensis* is distinct from *A. jiangxiense* in its larger conidia (globose or subglobose, 9.0–13.5 × 7.0–12.0 μm in *A. camelliae-sinensis* vs. surface view 7.5–10.0 μm diam, side view 4.5–7.0 μm diam in *A. jiangxiense*) and conidiogenous cell arrangement (aggregated irregularly on hyphae vs. scattered on hyphae in *A. jiangxiense*) and distinct from *A. obovatum* in the lack of obovoid conidia (see the note under *A. obovatum*).

**Figure 4.**
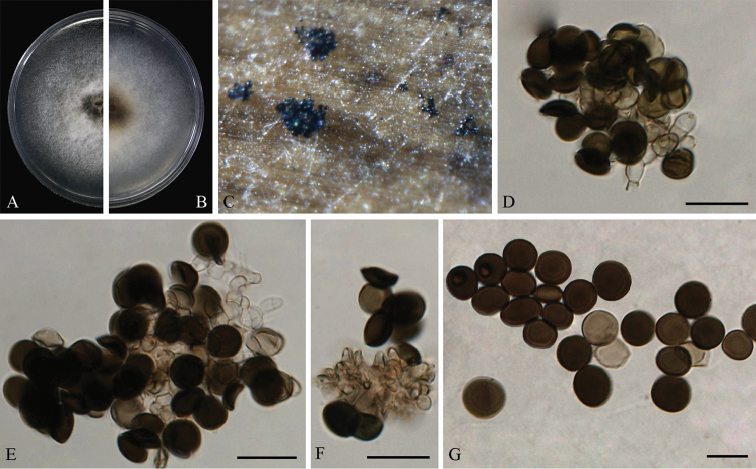
*Arthrinium camelliae-sinensis* (from ex-holotype strain CGMCC 3.18333) **A–B** 7 d old cultures on PDA **C** Colony on MEA with bamboo leaves producing conidia masses **D–F** Conidiogenous cells giving rise to conidia **G** Conidia. Scale bars = 10 μm.

### Arthrinium dichotomanthi

Taxon classificationFungiXylarialesApiosporaceae

M. Wang & L. Cai sp. nov.

824908

Figure 5

#### Type.

CHINA, Chongqing, on *Dichotomanthus tristaniaecarpa*, 20 Dec. 2012, L. Cai, (holotype: HMAS 247185; culture ex-type: CGMCC 3.18332 = LC4950).

#### Etymology.

Named after the host from which it was isolated.

#### Description.

Hyphae hyaline, branched, septate, 1.5–5.0 μm diam. Conidiophores reduced to conidiogenous cells. Conidiogenous cells erect, aggregated in clusters on hyphae, hyaline to pale brown, smooth, doliiform to clavate or lageniform, 5.5–11.0 × 3.0–5.0 μm (*x̄* = 7.9 ± 1.4 × 4.0 ± 0.5, n = 30). Conidia brown to dark brown, smooth to finely roughened, globose, subglobose to lenticular, with a longitudinal germ slit, 9.0–15.0 × 6.0–12.0 μm (*x̄* = 12.0 ± 1.4 × 8.5 ± 1.1, n = 50).

#### Culture characteristics.

On PDA, colonies umbonate, margin irregular, with sparse aerial mycelia. Colonies creamy-white to greyish without patches reverse, reaching 9 cm in 7 days at 25 °C. On MEA, colonies flat, spreading, surface and reverse pale luteous.

#### Other specimens.

CHINA, Chongqing, on *Dichotomanthus tristaniaecarpa*, 20 Dec. 2012, L. Cai, living culture LC8175 = WM529; ibid. living culture LC8176 = WM 530.

#### Notes.

Three strains representing *A. dichotomanthi* formed a distinct clade closely related to *A. phaeospermum* (Corda) M.B. Ellis (99% sequence similarity in ITS; 96% in TUB2; 96% in TEF1), *A. serenense* Larrondo & Calvo (99% sequence similarity in ITS; 95% in TUB2) and *A. saccharicola* F. Stevens (99% sequence similarity in ITS; 95% in TUB2; 97% in TEF1). *Arthrinium dichotomanthi* differs from *A. phaeospermum* and *A. saccharicola* in its larger conidia (globose or subglobose, 9.0–15.0 × 6.0–12.0 μm in *A. dichotomanthi* vs. surface view (9–)10(–12) μm diam, side view 6–7 μm diam in *A. phaeospermum*, surface view (7–)8–9(–10) μm diam, side view (4–)5(–6) μm diam in *A. saccharicola*) and from *A. serenense* by the absence of odour on the MEA colony (Larrondo 1990).

**Figure 5.**
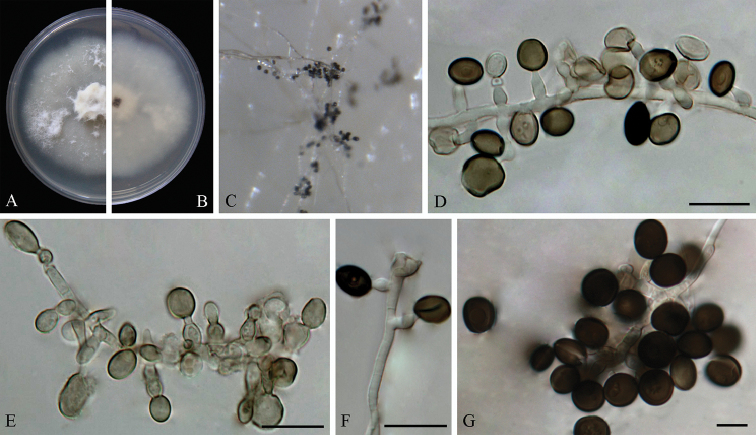
*Arthrinium dichotomanthi* (from ex-holotype strain CGMCC 3.18332) **A–B** 7 d old cultures on PDA **C** Colony on MEA producing conidia masses **D–F** Conidiogenous cells giving rise to conidia **G** Conidia. Scale bars = 10 μm.

### Arthrinium guizhouense

Taxon classificationFungiXylarialesApiosporaceae

M. Wang & L. Cai sp. nov.

824909

Figure 6

#### Type.

CHINA, Guizhou Province, from the air in karst cave, 23 Jul. 2014, Z.F. Zhang, (holotype: HMAS 247188; culture ex-type: CGMCC 3.18334 = LC5322).

#### Etymology.

Named after the province where type was collected, Guizhou province.

#### Description.

Hyphae hyaline, branched, septate, 1.5–5.0 μm diam. Conidiophores reduced to conidiogenous cells. Conidiogenous cells erect, aggregated in clusters on hyphae, pale brown, smooth, subglobose, ampulliform or doliiform, 3.5–8.0 × 3.0 – 4.5 μm (*x̄* =5.1 ± 1.08 × 3.7 ± 0.49, n = 30). Conidia dark brown to black, smooth to finely roughened, globose or subglobose, occasionally elongated to ellipsoidal, with a longitudinal, hyaline, thin, germ slit, 5.0–7.5 × 4.0–7.0 μm (*x̄* = 6.1 ± 0.5 × 5.5 ± 0.6, n = 50).

#### Culture characteristics.

On PDA, colonies flat, woolly, margin circular, with moderate aerial mycelia, surface initially white, becoming greyish and reverse with black patches, reaching 9 cm in 9 days at 25 °C. On MEA, surface dirty white with patches of olivaceous-grey and reverse greyish.

#### Other specimens examined.

CHINA, Guizhou Province, from the air in karst cave, 23 Jul. 2014, Z.F. Zhang, living culture LC5318.

#### Notes.

*Arthrinium guizhouense* is closely related to *A. sacchari* (Speg.) M.B. Ellis (99% sequence similarity in ITS; 99% in TUB2; 94% in TEF1). Morphologically, *A. guizhouense* and *A. sacchari* are very similar in conidial size, but *A. guizhouensis* produces relatively shorter conidiogenous cells (3.5–8.0 μm in *A. guizhouense* vs. 5–12 μm in *A. sacchari*).

**Figure 6.**
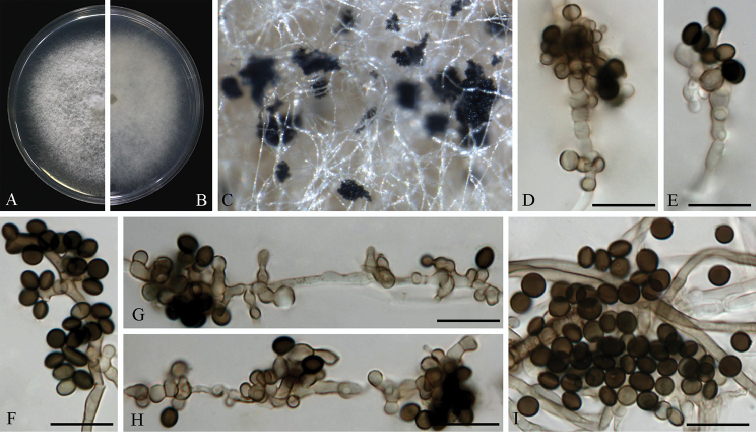
*Arthrinium guizhouense* (from ex-holotype strain CGMCC 3.18334) **A–B** 6 d old cultures on PDA **C** Colony on MEA producing conidia masses **D–H** Conidiogenous cells giving rise to conidia **I** Conidia. Scale bars = 10 μm.

### Arthrinium jiangxiense

Taxon classificationFungiXylarialesApiosporaceae

M. Wang & L. Cai sp. nov.

824910

Figure 7

#### Type.

CHINA, Jiangxi Province, on *Maesa* sp., 05 Sept. 2013, Y.H. Gao, (holotype: HMAS 247183; culture ex-type: CGMCC3.18381 = LC4577).

#### Etymology.

Named after the province where the most strains of this species were collected, Jiangxi.

#### Description.

Hyphae hyaline, branched, septate, 1.5–5.0 μm diam. Conidiophores reduced to conidiogenous cells. Conidiogenous cells erect, scattered or aggregated in clusters on hyphae, hyaline to pale brown, smooth, ampulliform, 6.0–15.0 × 2.5–5.0 μm (*x̄* = 9.7 ± 2.6 × 3.7 ± 0.6, n = 30), apical neck 2.5–6.0 μm long, basal part 3.0–9.0 μm long. Conidia brown, smooth to finely roughened, granular, globose to ellipsoid in surface view, 7.5–10.0 μm diam (*x̄* = 8.7 ± 0.6, n = 50), lenticular in side view, with longitudinal, pale germ slit, 4.5–7.0 μm diam (*x̄* = 5.8 ± 0.6, n = 50). Sterile cells forming on solitary loci on hyphae, brown, finely roughened, subcylindrical to clavate.

#### Culture characteristics.

On PDA, colonies flat, woolly, margin circular, with sparse aerial mycelia, initially white, becoming greyish due to sporulation, reaching 9 cm in 10 days at 25 °C, on MEA, sienna with patches of luteous, reverse luteous to sienna.

#### Other specimens examined.

CHINA, Hunan Province, on bamboo, 22 Sept. 2010, L. Cai, living culture LC2831; Jiangxi Province, on *Phyllostachys* sp., 05 Sept. 2013, Y.H. Gao, living culture LC4494; on *Phyllostachys* sp., 22 Apr. 2013, Q. Chen, living culture LC4993; ibid. living culture LC4497; ibid. living culture LC5001; ibid. living culture LC5004; on *Imperata cylindrical*, 22 Apr. 2013, Q. Chen, living culture LC5015; on *Maesa* sp., 05 Sept. 2013, Y.H. Gao, living culture LC4541; on *Machilus* sp., 05 Sept. 2013, Y.H. Gao, living culture LC4547; on *Camellia sinensis*, 05 Sept. 2013, Y.H. Gao, living culture LC4578; on bamboo, 01 Jul. 2016, J.E. Huang, living culture LC7104; ibid. living culture LC7154; ibid. living culture LC7156; ibid. living culture LC7275.

#### Notes.

Two strains representing *Arthrinium jiangxiense* clustered in a well-supported clade and appeared closely related to *A. camelliae-sinensis* (97% sequence similarity in ITS; 94% in TUB2; 94% in TEF1). While *A. jiangxiensis* is distinct from *A. camelliae-sinensis* in its smaller conidia (surface view 7.5–10.0 μm diam, side view 4.5–7.0 μm diam in *A. jiangxiensis* vs. globose or subglobose, 9.0–13.5 × 7.0–12.0 μm in *A. camelliae-sinensis*) and conidiogenous cell arrangements (conidiogenous cells scattered on hyphae vs. aggregated irregularly on hyphae in *A. jiangxiense*).

**Figure 7.**
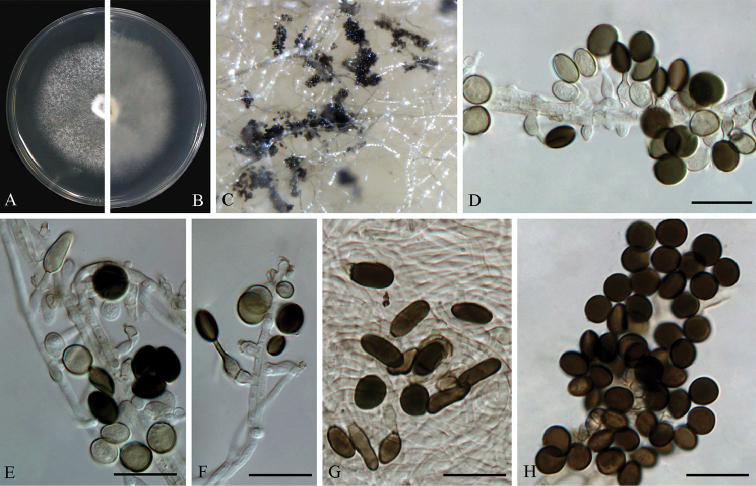
*Arthrinium jiangxiense* (from ex-holotype strain CGMCC 3.18381) **A–B** 5 d old cultures on PDA **C** Colony on MEA producing conidia masses **D–F** Conidiogenous cells giving rise to conidia **G** Elongated conidia **H** Conidia. Scale bars = 10 μm.

### Arthrinium obovatum

Taxon classificationFungiXylarialesApiosporaceae

M. Wang & L. Cai sp. nov.

824911

Figure 8

#### Type.

CHINA, Chongqing, on *Lithocarpus* sp., 20 Dec. 2012, L. Cai, (holotype: HMAS 247184; culture ex-type: CGMCC 3.18331 = LC4940).

#### Etymology.

Referring to the production of the large obovoid conidia.

#### Description.

Hyphae hyaline to pale brown, branched, septate, 1.5–5.0 μm diam. Conidiophores reduced to conidiogenous cells. Conidiogenous cells erect, aggregated in clusters on hyphae, pale brown, smooth, subcylindrical or clavate, 5.5–13.5 × 2.5–5.0 μm (*x̄* = 8.7 ± 2.4 × 3.6 ± 0.6, n = 30). Conidia dark brown, roughened, globose to subglobose, 11.0–16.5 μm (*x̄* = 13.8 ± 1.5, n = 50) in diam.; obovoid, 16.0–31.0 × 9.0–16.0 μm (*x̄* = 23.0 ± 2.7 × 12.7 ± 1.4, n = 50), occasionally elongated to ellipsoidal.

#### Culture characteristics.

On PDA, colonies flat, spreading, margin circular, initially white, becoming olivaceous-grey on surface, reverse smoke-grey with patches of olivaceous grey, reaching 9 cm in 7 days at 25 °C. On MEA, surface olivaceous grey in the central and luteous around, reverse with patches of olivaceous grey.

#### Other specimens examined.

CHINA, Chongqing, on *Lithocarpus* sp., 20 Dec. 2012, L. Cai, living culture LC8177; ibid. living culture LC8178.

#### Notes.

**Figure 8.**
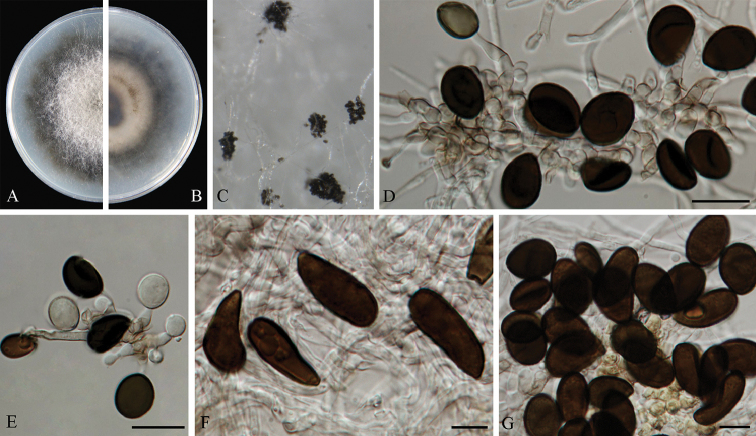
*Arthrinium obovatum* (from ex-holotype strain CGMCC 3.18331) **A–B** 7 d old cultures on PDA **C** Colony on MEA producing conidia masses **D–E** Conidiogenous cells giving rise to conidia **F** Obovoid conidia **G** Globose to subglobose conidia. Scale bars = 10 μm.

*Arthrinium obovatum* is the only species that produces obovoid conidia (Figure 8F) in this genus, a character distinctly different from other species (Ellis 1965, 1976, Gjaerum 1967, Pollack and Benjamin 1969, Hudson et al. 1976, Calvo and Guarro 1980, Khan and Sullia 1980, Samuels et al. 1981, von Arx 1981, Koskela 1983, Kirk 1986, Larrando and Calvo 1990, 1992, Müller 1992, Bhat and Kendrick 1993, Hyde et al. 1998, Jones et al. 2009, Singh et al. 2012, Crous et al. 2013, 2015, Sharma et al. 2014, Senanayake et al. 2015, Senanayake et al. 2015, Hyde et al. 2016, Dai et al. 2016a, b).

### Arthrinium pseudoparenchymaticum

Taxon classificationFungiXylarialesApiosporaceae

M. Wang & L. Cai sp. nov.

824912

Figure 9

#### Type.

CHINA, Guangdong Province, on bamboo, Jul. 2016, D.W. Xiao, (holotype: HMAS 247189; culture ex-type: CGMCC 3.18336 = LC7234).

#### Etymology.

Referring to the pseudoparenchymatous hyphae.

#### Description.

Hyphae hyaline to pale brown, branched, septate, 1.5–5.0 μm diam., pseudoparenchymatous. Conidiophores aggregated in hyaline to light brown sporodochia, smooth, usually unbranched, up to 40 μm long, 3–6 μm width. Conidiogenous cells hyaline to pale yellow, smooth to finely roughened, subcylindrical to doliiform, 8.0–18.5 × 3.0–8.5μm (*x̄* = 13.7 ± 3.2 × 5.4 ± 1.2, n = 30). Conidia pale to dark brown, smooth, finely guttulate, globose to subglobose, 13.5–27.0 × 12.0–23.5 μm (*x̄* = 20.2 ± 2.5 × 17.1 ± 2.4, n = 50). Sometimes lobed or dentate, polygonal or irregular in surface view.

#### Culture characteristics.

On PDA, colonies flat, spreading, margin circular, with moderate aerial mycelia, initially white, becoming grey on surface, reverse smoke-grey without patches, reaching 9 cm in 8 days at 25 °C. On MEA, surface pale luteous to grey with abundant mycelia, reverse greyish without patches.

#### Other specimens examined.

CHINA, Guangdong Province, on bamboo, Jul. 2016, D.W. Xiao, living culture LC8173; ibid. living culture LC8174.

#### Notes.

*Arthrinium pseudoparenchymaticum* is closely related to *A. hyphopodii* (94% sequence similarity in ITS), but differs in its much larger conidia (13.5–27.0 × 12.0–23.5 μm vs. 5–10 × 4–8 μm), the absence of hyphopodia and the presence of dentate conidia.

**Figure 9.**
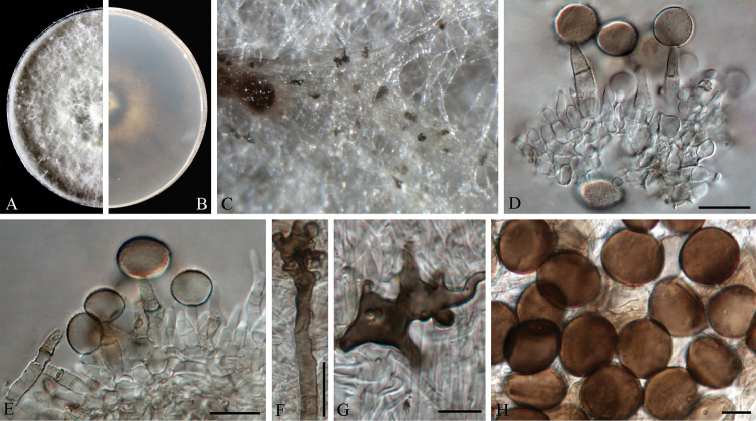
*Arthrinium pseudoparenchymaticum* (from ex-holotype strain CGMCC 3.18336) **A–B** 8 d old cultures on PDA **C** Colony on MEA producing conidia masses **D–E** Conidiogenous cells giving rise to conidia **F–G** Dentate conidia **H** Globose conidia. Scale bars = 10 μm.

### Arthrinium subroseum

Taxon classificationFungiXylarialesApiosporaceae

M. Wang & L. Cai sp. nov.

824913

Figure 10

#### Type.

CHINA, Jiangxi Province, on bamboo, 1 Jul. 2016, J.E. Huang, (holotype: HMAS 247190; culture ex-type: CGMCC3.18337 = LC7292).

#### Etymology.

Named after the colour of colony on PDA, pinkish.

#### Description.

Hyphae hyaline to pale brown, branched, septate, 1.5–6.0 μm diam. Conidiophores hyaline to pale brown, smooth, erect or ascending, simple, flexuous, subcylindrical, clustered in groups. Conidiophores aggregated in brown sporodochia, smooth, hyaline to brown, up to 20 μm long, 2–4.5 μm width. Conidiogenous cells pale brown, smooth, doliiform to subcylindrical, 3.0–6.5 × 2.0–5.0 μm (*x̄* = 4.7 ± 1.2 × 3.7 ± 0.9, n = 30). Conidia pale brown to dark brown, smooth, globose to subglobose or ellipsoidal, 12.0–17.5 × 9.0–16.0 μm (*x̄* = 14.9 ± 1.4 × 11.8 ± 1.8, n = 50).

#### Culture characteristics.

On PDA, colonies flat, spreading, margin circular, with moderate aerial mycelia, initially white, becoming light pink on surface, reverse peach-puff without patches, reaching 10 cm in 8 days at 25 °C. On MEA, surface blackish-green with abundant mycelia, reverse with patches of greyish.

#### Other specimens.

CHINA, Jiangxi Province, on bamboo, 1 Jul. 2016, J.E. Huang, living culture LC7215; ibid. living culture LC7291.

#### Notes.

Three strains representing *A. subroseum* clustered in a well-supported clade, closely related to *A. garethjonesii* (94% sequence similarity in ITS) and *A. bambusae* (98% sequence similarity in ITS; 92% in TUB2; 96% in TEF1). However, *A. subroseum* differs from *A. bambusae* in the morphology of conidiophores (erect or ascending, clustered in groups in *A. subroseum* vs. reduced to conidiogenous cells in *A. bambusae*). *Arthrinium subroseum* is not morphologically comparable to *A. garethjonesii*, whose asexual morph is undetermined (Dai et al. 2016b).

**Figure 10.**
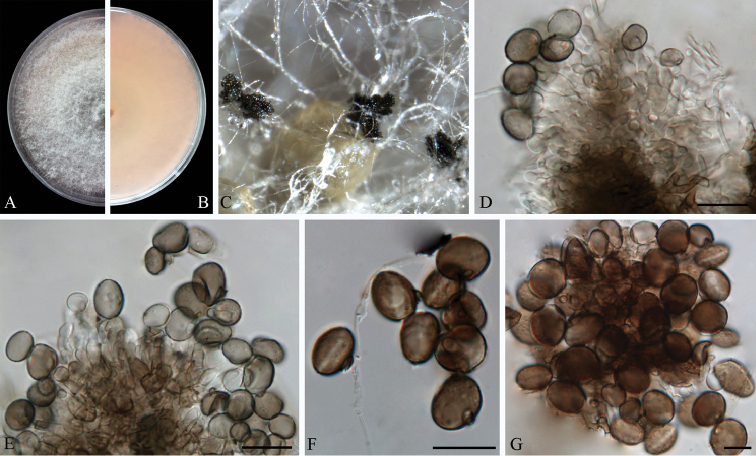
*Arthrinium subroseum* (from ex-holotype strain CGMCC3.18337) **A–B** 10 d old cultures on PDA **C** Colony on MEA producing conidia masses **D–E** Conidiogenous cells giving rise to conidia **F–G** Conidia. Scale bars = 10 μm.

## Discussion

*Arthrinium*, *Cordella* and *Pteroconium* share similar morphological characters, e.g. basauxic conidiophores with terminal and intercalary polyblastic conidiogenous cells and brown, unicellular conidia with a pallid germ slit (Ellis 1971, Hyde et al. 1998). Crous et al. (2013) reduced both *Cordella* and *Pteroconium* as generic synonyms of *Arthrinium* based on molecular phylogenetic data and regarded traditionally applied morphological characters in distinguishing these genera as phylogenetically insignificant. This study added eight novel species and our data are in good accordance with that of Crous et al. (2013). For example, *A. pseudoparenchymaticum* is sporodochial and pseudoparenchymatous, which would be classified as *Pteroconium* in the traditional taxonomy. However, the multi-locus (ITS, TEF1 & TUB2) tree (Figure. 1) shows that *A. pseudoparenchymaticum* is phylogenetically distant from *A. pterospermum* (syn. *P. pterospermum*, the type of “*Pteroconium*”).

Currently there are 70 recognised species in *Arthrinium* (Index Fungorum), occurring on a wide variety of both living and decaying plant materials. It is noteworthy that *Arthrinium* species showed distinct preference for growing on two graminaceous families, Poaceae and Cyperaceae, amongst which, *Bambusa* (Poaceae) and *Carex* (Cyperaceae) are two of the most common host genera for *Arthrinium* species. For example, seven species have been recorded from *Carex* spp., i.e. *A. austriacum* Petr. (1959), *A. caricicola* Kunze (1817), *A. globosum* Koskela (1983), *A. kamtschaticum* Tranzschel & Woron (1914), *A. morthieri* Fuckel (1870), *A. muelleri* Ellis (1976) and *A. naviculare* Rostr. (1886). Bamboo has been widely known as a favourable host for *Arthrinium*, e.g. *A. hyphopodii*, *A. longistromum*, *A. subglobosum*, *A. thailandicum* and *A. yunnanum* (Senanayake et al. 2015, Dai et al. 2016). In this study, three new species (*A. bambusae*, *A. subroseum* and *A. pseudoparenchymaticum*) were also isolated from bamboo. In addition, three species (*A. arundinis*, *A. guizhouense*, and *A. rasikravindrii*) were isolated from air and soil from karst caves, where have been shown to encompass a high fungal diversity (Jiang et al. 2017, Zhang et al. 2017).

**Figure 11.**
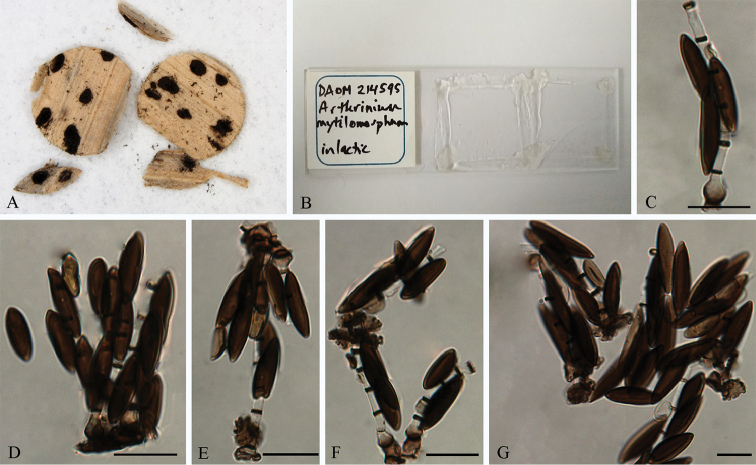
*Arthrinium mytilomorphum* (from holotype DAOM 214595) **A–B** Overview of the type specimen **C–F** Conidiogenous cells giving rise to conidia **G** Conidia. Scale bars = 10 μm.

In addition to the *Arthrinium* species from China, we also tried to resolve the phylogenetic status of *Arthrinium mytilomorphum* Bhat & W.B. Kendr. (Bhat and Kendrick 1993) in the current study. DNA extraction from the type specimen of *A. mytilomorphum* (DAOM 214595) was prohibited but DAOM provided a DNA sample. Unfortunately, we only managed to obtain an ITS sequence from this DNA sample, while the amplifications of all other protein coding genes were unsuccessful. The ITS phylogenetic tree (not shown here) shows that *A. mytilomorphum* is closely related to *A. subroseum* (99 % sequence similarity in ITS), while the morphology of these two species are very different from each other. Conidia of *A. mytilomorphum* are dark brown, fusiform or navicular, measuring 20–30 × 6–8.5 μm, slightly bowed down and asymmetric (Figure 11), while those of *A. subroseum* are pale brown to dark brown, globose or subglobose, measuring 12–17.5 × 9–16 μm.

Teleomorph-typified genus *Apiospora* was treated as a synonym of anamorph-typified genus *Arthrinium* on the basis that *Arthrinium* is older and more commonly used in literature (Crous et al. 2013). However, only three of the 58 recorded *Apiospora* species have been properly linked to their known *Arthrinium* counterparts, i.e. *Arthrinium hysterinum* (syn. *Ap. bambusae*) (Sivanesan 1983, Kirk 1986); *Arthrinium arundinis* (syn. *Ap. montagnei*) (Hyde 1998); *Arthrinium sinense* (syn. *Ap. sinensis*) (Réblová et al. 2016). In addition, molecular data of only four *Apiospora* species (*Ap. bambusae*, *Ap. montagnei*, *Ap. setosa* and *Ap. sinensis*) are available, in which only *A. bambusae* and *A. sinensis* have type-derived sequences. A comprehensive taxonomic revision of this taxonomic group awaits fresh collection and epitypification of many *Apiospora* species and, based on which, phylogenetic links with *Arthrinium* species could be established.

## Supplementary Material

XML Treatment for Arthrinium bambusae

XML Treatment for Arthrinium camelliae-sinensis

XML Treatment for Arthrinium dichotomanthi

XML Treatment for Arthrinium guizhouense

XML Treatment for Arthrinium jiangxiense

XML Treatment for Arthrinium obovatum

XML Treatment for Arthrinium pseudoparenchymaticum

XML Treatment for Arthrinium subroseum
